# Uncommon Nasal Mass Presentation: A Radiological Case Series

**DOI:** 10.3390/jpm14121145

**Published:** 2024-12-09

**Authors:** Antonio Lo Casto, Francesco Lorusso, Ettore Palizzolo, Federico Sireci, Francesco Dispenza, Manfredi De Angelis, Angelo Immordino, Salvatore Gallina, Francesco Bencivinni

**Affiliations:** 1 Radiological Sciences Section, Department of Biomedicine, Neuroscience and Advanced Diagnostics, University of Palermo, AOUP “Paolo Giaccone”, Via del Vespro 129, 90127 Palermo, Italy; antonio.locasto@unipa.it (A.L.C.); ettore.palizzolo@unipa.it (E.P.); manfredi.deangelis@unipa.it (M.D.A.); salvatore.gallina@unipa.it (S.G.); francesco.bencivinni@policlinico.pa.it (F.B.); 2 Otorhinolaryngology Section, Department of Biomedicine, Neuroscience and Advanced Diagnostics, University of Palermo, AOUP “Paolo Giaccone”, Via del Vespro, 129, 90127 Palermo, Italy; francesco.lorusso@policlinico.pa.it (F.L.); francesco.dispenza@unipa.it (F.D.); 3 Otorhinolaryngology Section, Department of Precision Medicine in Medical, Surgical and Critical Care (Me.Pre.C.C), University of Palermo, Via del Vespro 129, 133, 90127 Palermo, Italy; federico.sireci@unipa.it; 4 Dental and Maxillofacial Radiology Section, AOUP “Paolo Giaccone”, Via del Vespro, 129, 90127 Palermo, Italy

**Keywords:** nose, masses, tumors, maxillary sinus, radiology, CT, CBCT, MRI

## Abstract

**Background:** Nasal and paranasal sinus masses can arise from a wide range of conditions, both benign and malignant, as well as congenital or acquired. Diagnosing these masses is often challenging, requiring a combination of nasal endoscopy, imaging studies, and histopathological analysis. Initial imaging frequently involves computed tomography or cone beam computed tomography (CBCT) to evaluate the bony anatomy of the nasal cavity and surrounding sinuses, while magnetic resonance imaging (MRI) is typically used for detailed assessment of soft tissues and to aid in differential diagnosis when the findings are inconclusive. **Methods:** This review examines nasal masses evaluated using CT, CBCT, and MRI, highlighting key imaging features that may assist in differential diagnosis. **Results:** For non-neoplastic lesions, examples include conditions such as rhinoliths, inverted mesiodens, and septal mucoceles. Benign and borderline tumors discussed encompass lobular capillary hemangioma, inverted papilloma, septal osteoma, chondromesenchymal hamartoma, hemangioma, hemangiopericytoma, antrochoanal polyp, sinonasal angiofibroma, ossifying fibroma, and lipoma. Malignant tumors addressed in this review include adenocarcinoma, esthesioneuroblastoma, non-Hodgkin lymphoma, melanoma, and sarcoma. **Conclusions:** Diagnosing nasal lesions represent a significant challenge for otolaryngologists. Imaging characteristics of nasal masses play a crucial role in narrowing down differential diagnoses before surgery. However, nasal endoscopy combined with biopsy remains the definitive diagnostic approach.

## 1. Introduction

The nasal cavity and paranasal sinuses are susceptible to a broad range of conditions, encompassing benign and malignant, congenital and acquired, as well as infectious and neoplastic ones. Diagnosing a nasal mass can be complex, as patients often present with diverse and nonspecific symptoms. A comprehensive diagnostic approach typically integrates nasal endoscopy, imaging studies, and histopathological evaluation, particularly for rare or unusual lesions. Computed tomography (CT) or cone beam computed tomography (CBCT) are commonly used as the first-line imaging techniques because of their effectiveness in assessing bony structures and surrounding anatomy. For cases requiring more detailed differentiation, magnetic resonance imaging (MRI) with an intravenous (i.v.) contrast medium administration is preferred due to its superior soft tissue resolution and ability to provide enhanced diagnostic insights. AI-related approaches, particularly deep learning, hold significant promise for the evaluation of sinonasal masses by CT and MRI. Applications include image acquisition and reconstruction, segmentation, classification and diagnosis, as well as prognosis prediction. Nevertheless, numerous challenges must be overcome before deep learning techniques can be widely implemented in routine radiological practice. The development and integration of AI-based methodologies require careful monitoring to ensure reliability, safety, and efficacy in clinical applications [1].

## 2. Materials and Methods

This study provides a concise review of nasal cavity diseases, supported by a retrospective case series of nasal masses evaluated at our institution from 2010 to 2023. The patients underwent evaluation using various imaging modalities, including multidetector CT, CBCT, and MRI, to identify key diagnostic features for differentiating nasal masses. Both male and female patients of all ages were included, provided that imaging strongly suggestive of a specific diagnosis was available. We excluded some cases with nonclinical and nondiagnostic outcomes. The findings were correlated with clinicopathological data, with all imaging results confirmed through histopathological analysis. This study outlines distinct imaging characteristics of benign and malignant nasal masses observed with CT/CBCT and MRI, both before and after the administration of intravenous contrast agents—iodinated contrast for CT and gadolinium-based contrast for MRI—to assess the vascular characteristics of the masses.

Non-neoplastic examples discussed include rhinoliths, inverted mesiodens, and septal mucoceles. This review also examines benign and borderline tumors such as lobular capillary hemangioma, inverted papilloma, septal osteoma, hamartoma, hemangioma, hemangiopericytoma, antrochoanal polyp, sinonasal angiofibroma, cemento-ossifying fibroma, and lipoma. Among malignant tumors, cases of adenocarcinoma, esthesioneuroblastoma, non-Hodgkin lymphoma, melanoma, and sarcoma are presented.

## 3. Pictorial Review

### 3.1. Non-Neoplastic Lesions

#### 3.1.1. Septal Mucocele

Mucoceles are benign and uncommon lesions, characterized by a cystic structure lined with epithelium and filled with typically sterile mucoid material. Their slow, expansive growth is attributed to a dynamic process involving bone resorption, erosion, and remodeling [2]. While the exact cause of mucoceles remains unclear, they are generally categorized into primary forms, which arise from cystic degeneration of the sinus mucosa, and secondary forms, which result from obstruction of the paranasal sinus ostia, leading to mucus accumulation [3]. The majority of paranasal mucoceles occur in the frontal sinuses (60%), followed by the ethmoid (20–30%), maxillary (10%), and sphenoid (2–3%) sinuses [4]. These lesions often remain asymptomatic until they extend into adjacent structures. Septal mucoceles are exceptionally rare and are typically observed in men around the age of 50. Common symptoms include nasal obstruction and headache [5]. Nasal endoscopy usually reveals a septal mucocele as a soft, bilateral swelling of the nasal septum, frequently affecting the upper region [2].

Histologically, mucoceles consist of mucoid fluid, respiratory epithelium, and inflammatory cells. Management depends on the nature of the lesion. Malignant lesions necessitate complete excision, including resection of all layers of the nasal septum. In contrast, benign mucoceles require histological typing to guide the appropriate surgical approach [6].

**Figure 1:**
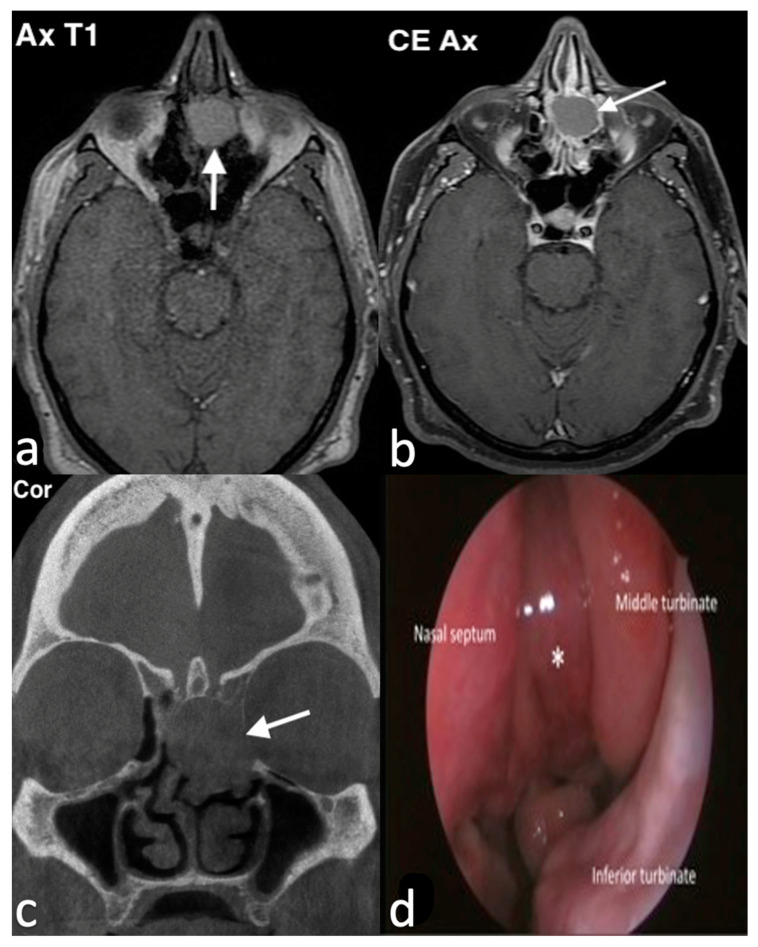
Septal mucocele in a 49-year-old man who had been experiencing long-standing bilateral nasal obstruction. Axial T1 MR image (**a**) shows an oval mass with well-defined margins, high intensity content; due to fluid-proteinaceous content, in the middle-upper third of nasal cavity the mass causes partial nasal obstruction in both nasal cavities. On axial T1, fat saturation after intravenous Gd (**b**), the lesion shows peripheral wall enhancement. On coronal CBCT (**c**), the nasal septum bone is expanded by the mass (arrow), laterally displacing the middle-left turbinate. Endoscopic view (**d**) of the left nasal cavity shows the mucocele (asterisk) between nasal septum and middle left turbinate.

Imaging plays a pivotal role in the evaluation of mucoceles and their effect on surrounding structures. CT and MRI are the first-line diagnostic tools [7]. These modalities are also essential for differential diagnosis. CT scans typically show mucoceles as smooth, rounded, fluid-filled lesions, often accompanied by bone erosion. On MRI, they appear hypointense on T1 images, although hyperintensity may be seen in cases with higher protein content in the mucoid secretions, and hyperintense on T2 images (Figure 1). Infected mucoceles, or mucopyoceles, may show rim enhancement on imaging, with variable signal intensities on both T1 and T2 sequences [8].

The standard treatment involves marsupialization or complete excision, both of which are curative and associated with minimal risk of complications. Notably, no recurrences have been reported in the literature [5].

Given their potential for complications such as bone erosion, mucoceles should be considered in the differential diagnosis of patients presenting with nasal obstruction, headache, and nasal cavity masses. Early identification and treatment are critical to prevent further complications [5].

#### 3.1.2. Rinolith

Foreign bodies are commonly found in children and individuals with intellectual disabilities, as these groups are more likely to insert objects into the nasal cavities. The prolonged presence of foreign bodies in the nasal cavities can lead to chronic inflammation, which facilitates the gradual deposition of mineral salts, eventually resulting in the formation of rhinoliths [9]. In many cases, foreign bodies may remain asymptomatic for many years [10]. Symptoms typically arise in adults presenting with persistent purulent rhinorrhea, unilateral nasal congestion, or headaches [9,10]. However, the clinical presentation can vary depending on the size and location of the rhinolith [10].

Rhinoliths are most commonly located on the floor of the nasal cavity, between the inferior turbinate and the nasal septum [9,11]. The precise location of the rhinolith is likely influenced by the anatomical features of the nasal cavity [9]. In some cases, rhinoliths may grow large enough to cause erosion of the nasal septum, affecting both nasal cavities [10,12]. Notably, there have been reports of contralateral septal deviation due to the mass effect of the rhinolith [13,14].

**Figure 2:**
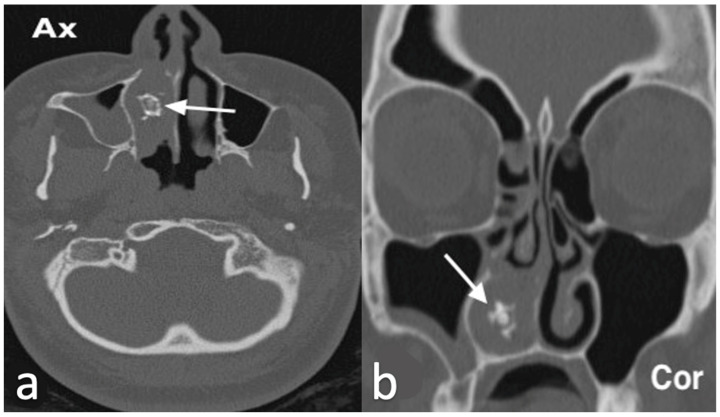
Rinolith in a male patient with history of right nasal obstruction, worsening with fetid purulent, both front and rear, rhinorrhea for about 2 years. On axial and coronal CT (**a**,**b**), the right nasal cavity is obstructed by an irregular calcified, hyperdense lesion (arrow) with mucosal thickening and, due to the mass effect, the medial wall of the right maxillary sinus is slightly compressed and laterally bowed. Nasal endoscopy confirmed the presence of a huge rinolith with wooden consistency in the right nasal cavity adhering to the nasal septum.

Histologically, rhinoliths appear as structures composed of concentric laminations of calcified mucinous material [10,15]. Nasal endoscopy and CT are key tools for diagnosing rhinoliths (Figure 2). CT is particularly useful for detecting calcium deposits, which are hyperdense on images, evaluating surrounding structures, and assisting in the differential diagnosis [13]. Treatment typically involves endoscopic surgical removal of the rhinolith, followed by postoperative antibiotic treatment [14,16]. The differential diagnosis should also consider other conditions that cause calcific deposits, such as ossifying fibroma, odontomas, osteomas, and osteosarcomas [14,16].

#### 3.1.3. Inverted Mesiodens

Mesiodens, the most common type of supernumerary tooth, has a prevalence of 0.15–5% [11]. It is typically asymptomatic and often discovered incidentally during radiological examinations of the upper jaw. Mesiodens can present in a normal orientation or an inverted position, with the crown directed toward the nasal cavity and the root positioned toward the oral cavity. The eruption of a mesiodens into the sinonasal cavities is an extremely rare phenomenon, occurring in only 0.1–1% of the population [11].

When a mesiodens erupts into the sinonasal cavities, symptoms may include nasal discharge, unilateral nasal obstruction, epistaxis, and facial pain [12].

**Figure 3:**
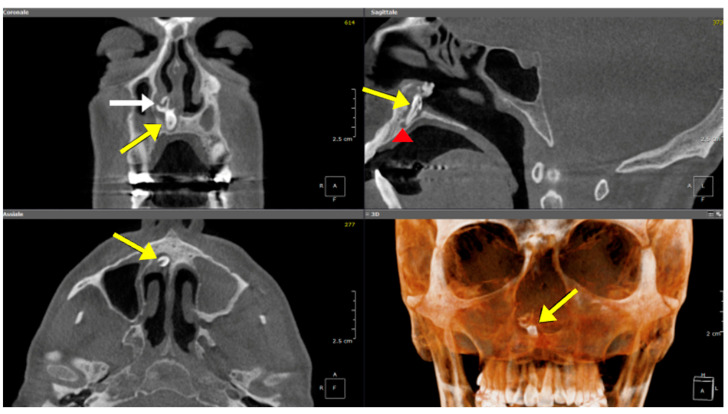
Inverted mesiodens in a 60-year-old male with unilateral nasal obstruction and blood discharge from his nose submitted to a CBCT. CBCT shows an inverted supernumerary tooth (inverted mesiodens) erupted into the right nasal cavity. The inverted mesiodens (yellow arrow), was carious with a periapical lesion (red arrowhead) and its crown was surrounded by reactive tissue with a rhinolith (white arrow).

From a histopathological perspective, the inverted mesiodens exhibits characteristics indistinguishable from those of a tooth [11]. CBCT is the preferred diagnostic tool, as it allows for precise localization and aids in planning the surgical approach (Figure 3).

### 3.2. Benign and Borderline Tumors

#### 3.2.1. Lobular Capillary Hemangioma

Lobular capillary hemangioma (LCH) is a benign lesion characterized by an elevated, capillary-rich lesion, commonly found on the mucous membranes of the oral cavity and nasal region [17]. LCH can occur at any age, with an average onset around the fourth decade of life, and it shows no significant gender predilection [18].

The exact mechanism behind LCH development is not fully understood, but factors such as trauma, hormonal influences, and underlying microscopic arteriovenous malformations are thought to play a role. LCH is most frequently observed in the anterior nasal cavity, particularly in Little’s area or on the tips of the turbinates, suggesting that trauma may be a contributing factor in its pathogenesis [19].

The exact etiopathology of LCH remains unclear. Nevertheless, factors such as pregnancy, hormonal imbalances, the use of oral contraceptives, regional trauma and nasal picking are considered predisposing elements that may contribute to its development [18].

Clinically, LCH typically presents with symptoms such as unilateral epistaxis and/or nasal obstruction, often accompanied by headache or facial pain. Endoscopically, the lesion appears as a solitary, hypervascularized red mass, commonly located in the anterior nasal septum. For larger lesions, imaging is recommended to evaluate the full extent beyond what is visible through endoscopy.

**Figure 4:**
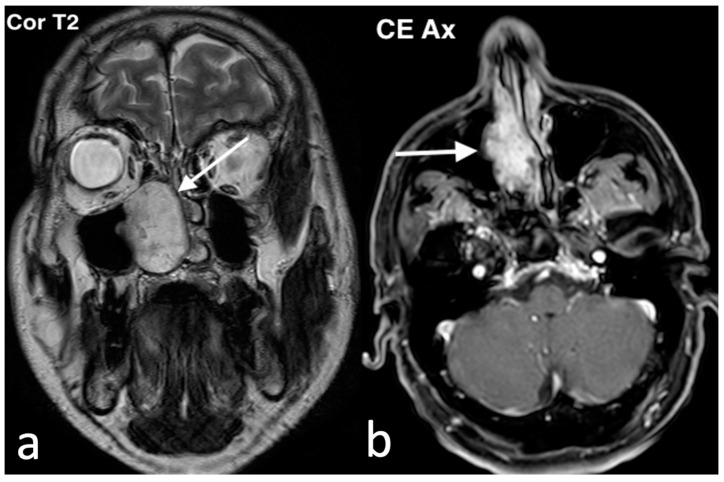
A lobular capillary hemangioma in a 61-year-old man with right nasal obstruction and epistaxis. On MR, an oval mass with smooth margins occupying most of the right nasal cavity, cranially attached to the ethmoid, is appreciable. On the T2 coronal image (**a**), the mass (arrow) shows a high signal intensity. On axial T1 fat saturation after intravenous Gd (**b**), the lesion (arrow) shows vivid enhancement with a unenhancing peripheral ring. Endoscopically, a slightly bleeding lesion, dark red in color, occupying almost the entire nasal cavity and implanted on the middle turbinate was evident.

Histologically, LCH consists of capillaries arranged in lobules separated by connective tissue, often infiltrated with numerous inflammatory cells [17]. On CT, LCH appears as a unilateral well-circumscribed soft tissue mass, without calcification, while MRI typically reveals T1 hypointensity and T2 hyperintensity with flow voids. Intense enhancement is noted following the administration of paramagnetic contrast agents with a hypointense, unhenancing rim (Figure 4). The mainstay treatment of LCH is endoscopic surgical excision [18].

#### 3.2.2. Chondromesenchymal Hamartoma

Nasal chondromesenchymal hamartoma (NCMH) is a rare benign tumor-like lesion that originates in the nasal cavity or paranasal sinuses, primarily affecting infants under one year of age. While initially thought to have a congenital or developmental origin, cases in adults with asymptomatic childhoods have been reported, potentially linked to chronic inflammation. Clinical presentations of NCMH include respiratory and feeding difficulties, rhinorrhea, epistaxis, visual disturbances, and otitis media. Importantly, no cases of malignant transformation have been documented to date [20].

Histologically, NCMH consists of a combination of mesenchymal components, including lobules of hyaline cartilage embedded within a fibrous matrix, sometimes interspersed with bone [20].

**Figure 5:**
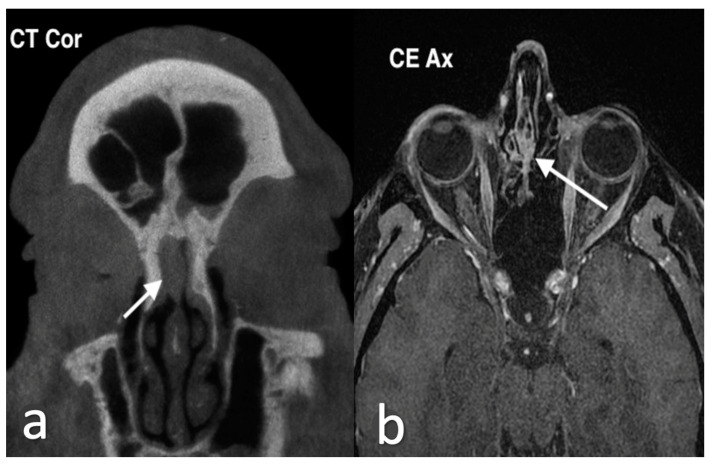
A nasal chondromesenchymal hamartoma in a patient with suspected rhinosinusitis. Coronal CBCT image (**a**) demonstrates, in the upper anterior third of the nasal septum, an oval lesion with well-defined margins and soft tissue density, extending in the upper third of both nasal cavities (arrow). No erosion of adjacent bony structure is visible. On MRI, in the T1 axial fat saturation image after intravenous Gd (**b**), a diffuse heterogeneous enhancement of the mass, with no enhancement of small cystic components, is appreciable (arrow).

Imaging is crucial for assessing the extent of NCMH and its potential involvement with surrounding structures, including the paranasal sinuses, orbit, and intracranial cavity. Despite its benign nature, NCMH can display aggressive characteristics such as bone erosion, thinning, displacement, and even intracranial extension through the cribriform plate. These features may mimic malignancy, although significant bony destruction is not typical of NCMH. On imaging, NCMH generally presents as a poorly defined, nonencapsulated mass, often containing cystic components. Calcifications are frequently observed [21]. MRI typically shows an inhomogeneous lesion with low signal intensity on T1 images and high signal intensity on T2 images, coupled with strongly inhomogeneous contrast enhancement. Enhancement patterns vary, ranging from mild enhancement to areas of prominent vascular proliferation, depending on the lesion composition and vascularity. While hypervascular NCMH has been documented, the degree of enhancement seen in hemangiomas or angiofibromas is variable. Cystic areas, which appear as bright signals on T2 images, may be extensive in some cases, causing the lesion to resemble a meningoencephalocele (Figure 5) [21].

The differential diagnosis includes conditions such as nasoethmoidal encephalocele, nasal glioma, rhabdomyosarcoma, lymphoma, and chondrosarcoma. Distinguishing NCMH from rhabdomyosarcoma and chondrosarcoma can be challenging on imaging, as the latter are often more destructive, poorly defined, and rapidly progressive [22]. Currently, complete surgical excision remains the preferred treatment for NCMH. Lesions confined to the nasal cavity are frequently amenable to minimally invasive endoscopic surgery.

A recent link has been identified between NCMH and mutations in the **DICER1** gene. Identifying this condition is crucial, as a germline **DICER1** mutation may increase the risk of developing other types of tumors [20].

#### 3.2.3. Ossifying Fibroma

Ossifying fibroma (OF) is a rare benign fibro-osseous tumor that predominantly affects women in their second to fourth decades of life. Although the mandible and maxilla are the most common sites for this lesion, it can occasionally occur in the nasal cavity or long bones.

The World Health Organization describes OF as being histologically defined by the substitution of normal bone with fibrous tissue, which includes varying quantities of mineralized material and cementum [23].

The tumor is often asymptomatic and discovered incidentally during imaging studies, such as CT or MRI, conducted for unrelated purposes.

Symptoms typically develop when the tumor expansive growth compresses adjacent structures. These symptoms may include sinusitis, nasal obstruction, cacosmia, facial swelling, maxillary pain, headache, fever, visual disturbances, exophthalmos, and proptosis, especially in cases involving the orbit. In the paranasal sinuses, OF can grow relatively uninhibited due to limited resistance from surrounding hard tissues, eventually causing noticeable clinical symptoms [24].

Diagnosis is frequently achieved through nasofibroscopy, which typically reveals a smooth, rounded mass with a hard consistency.

Histopathological analysis showed a proliferation of stromal spindle-shaped fibroblasts along with deposits of woven and lamellar bone containing scattered osteoblasts and osteocytes [23].

**Figure 6:**
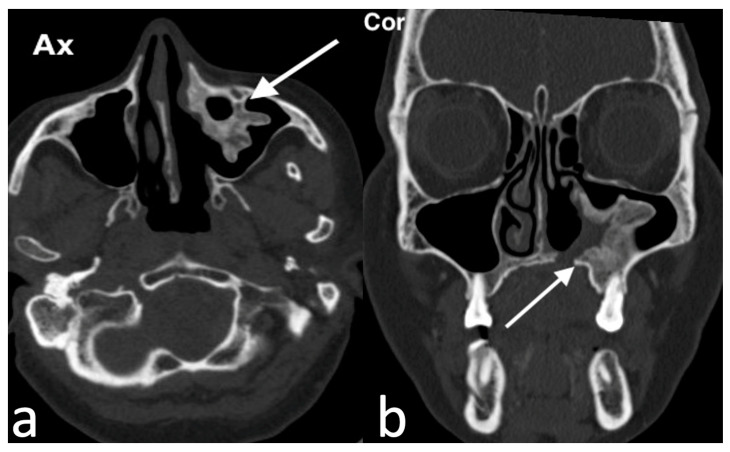
Ossifying fibroma in a 57-year-old woman with a clinical history of chronic sinusitis, who previously had undergone surgery of the left maxillary sinus. She was evaluated for the onset of painful and widespread left hemi-facial swelling, associated with fever, release of fetid purulent exudates and cacosmia. Rhinoscopy showed a lesion of bone consistency covered with normal-appearing mucosa occupying the lower portion of the left nasal cavity. Axial CT image (**a**) shows a lobulated and well-circumscribed mass, peripherally ossified (arrow); on coronal CT image (**b**), the mass (arrow) involves the lateral wall of left nasal cavity and caudally extents into the maxillary alveolar process in the periapical region of molar tooth 26. The patient underwent endoscopic re-excision of the lesion.

Imaging studies, particularly CT and MRI, are indispensable for accurate diagnosis and surgical planning. OF is characterized as a well-defined, hypodense, unilocular, expansile mass, without bony erosion and scattered radiopaque calcifications. Usually, there is a hyperdense, peripheral bony rim enclosing a fibrous soft tissue core. In more advanced stages, the lesion may appear completely ossified. On MRI, OF generally exhibits low to intermediate signal intensity on T1 images, and variable intensity on T2 images (Figure 6) [25].

Complete surgical excision is the preferred treatment, with the surgical approach tailored to the tumor’s size and location. However, recurrence remains a possibility, often due to incomplete removal of the lesion. Long-term follow-up with periodic nasal endoscopy and CT imaging is crucial to detect and manage recurrences early [24].

#### 3.2.4. Inverted Papilloma

Inverted papilloma (IP) is an uncommon benign epithelial tumor characterized by local aggressiveness, a tendency for recurrence, and occasional malignant transformation. Recurrences and metachronous carcinoma may develop even after extended periods. IP accounts for 0.5% to 4% of all primary nasal tumors and, typically diagnosed in individuals in their fifth or sixth decade of life, with a male-to-female ratio of 3:1 [26].

IP predominantly originates from the lateral nasal wall, often centered in the middle meatus. It may involve any paranasal sinus, with the maxillary sinus being the most commonly affected. Bilateral or multifocal IP cases have been documented but are exceedingly rare [26]. Clinical symptoms are nonspecific and mimic other nasal masses, typically including nasal obstruction and sinusitis-like presentations [27].

Histologically, the sinonasal mucosa consists of ciliated pseudostratified columnar epithelium, known as the Schneiderian membrane, with olfactory neuroepithelium lining the nasal cavity roof. Based on histomorphologic features, papillomas are classified into three subtypes: fungiform papilloma, oncocytic Schneiderian papilloma, and IP, the latter accounting for 62% of cases [27]. IP is distinguished by epithelial proliferation into the underlying connective tissue, which gives the lesion its characteristic “inverted” pattern. The architecture of this lesion is characterized by prominent downward endophytic growth, where round to elongated epithelial nests interconnect, forming a smooth outer contour. The epithelium itself is hyperplastic, ranging from 5 to 30 cell layers in thickness, and can be of various types, including squamous, transitional, or respiratory. The underlying stroma often shows signs of edema or chronic inflammation. Additionally, seromucinous glands in the lamina propria are typically decreased or may even be absent [27].

**Figure 7:**
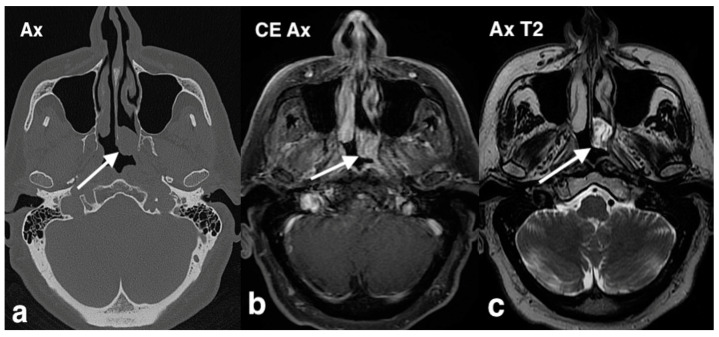
Inverted papilloma in an 83-year-old woman presenting with right nasal obstruction for 2 years associated with fronto-temporal headache and epistaxis. Endoscopically, a lobulated mass protruding in the left nasal cavity was found. The axial CT image (**a**) shows a mass (arrow) located in the posterior third of the left nasal cavity, between the nasal septum and medial pterygoid plate, and protruding posteriorly through the left choana in the nasopharynx. No bony erosion is visible. On MRI, in the T1 axial fat saturation image after intravenous Gd (**b**), the mass shows intense heterogeneous enhancement, with typical cerebroid pattern (arrow); on the T2 axial image, the mass shows inhomogeneous, cerebroid aspect, with high intensity, alternating hypointense and hyperintense striation throughout the mass (arrow) (**c**).

CT is generally the first imaging modality, as it effectively identifies bony changes. IP often leads to bone remodeling, including thinning, bowing, and resorption, without significant destruction. Two focal hyperostosis patterns are recognized on CT: cone-shaped and plaque-like. MRI provides additional diagnostic insights. IP usually appears iso- to hyperintense on T1 images and hypo- to iso-intense on T2 images relative to brain parenchyma or muscle. Post-gadolinium enhancement highlights the tumor borders, distinguishing it from obstructive secretions and aiding differentiation from other conditions, such as antrochoanal polyps, sinusitis, or mucoceles (Figure 7).

A hallmark MRI feature of IP is the “convoluted cerebriform pattern”, also referred to as striated, gyriform, or columnar morphology. This pattern reflects the histological structure: the compact epithelium corresponds to hypointense signals on T2 images and less enhancement post contrast, while subepithelial loose connective tissue appears hyperintense and enhances more strongly. These imaging features, combined with the absence of extensive bone erosion, allow for accurate discrimination of IP from malignant tumors, achieving diagnostic accuracy of up to 97.8% [26].

While cervical lymphadenopathy is uncommon in IP, its presence warrants evaluation for malignancy. Differential diagnoses include inflammatory polyps, esthesioneuroblastoma, and primary malignancies such as epithelial tumors and lymphoma. Inflammatory polyps are typically bilateral; unilateral cases require histological confirmation. Antrochoanal polyps, a rare form of inflammatory polyp, usually present unilaterally and exhibit peripheral enhancement due to inflammation on contrast-enhanced MRI. Esthesioneuroblastomas, arising from the superior nasal recess, are more likely to extend into the cribriform plate, display intense contrast enhancement, and show early intracranial involvement. Primary malignancies in the nasal cavity often feature bone destruction and rapid local invasion [27].

Historically, IP was managed with aggressive surgical techniques, such as medial maxillectomy via external incision. However, advances in imaging with CT and MRI have enabled precise preoperative planning, leading to a preference for minimally invasive endoscopic approaches in many cases.

#### 3.2.5. Osteoma

Osteoma is a benign bone lesion characterized by the proliferation of either compact or cancellous bone tissue, resulting in three distinct types: compact (also known as “ivory”), cancellous (mature) and mixed ivory/mature osteomas. These lesions are further categorized based on their origin: central osteomas, which develop from the endosteum; peripheral osteomas, arising from the periosteum; and extraskeletal osteomas, which occur within soft tissues such as muscle [28].

The exact cause of osteoma formation remains uncertain. While some experts regard osteomas as true neoplasms, others suggest they may be developmental anomalies. An additional hypothesis proposes a reactive origin, triggered by trauma or infection.

These tumors are generally slow-growing, asymptomatic, and often detected incidentally on imaging. When symptomatic, they can mimic sinusitis and may lead to obstructive complications. Tumor growth can block sinus ostia, disrupt mucociliary clearance, and result in localized symptoms associated with sinusitis, particularly in the frontal and ethmoid regions. Severe complications include the formation of a frontal mucocele, orbital compression, or intracranial extension [29]. Osteomas are classified as “giant” when their size reaches or exceeds 3 cm. The frontal sinus is most commonly affected, followed by the ethmoid and maxillary sinuses. Patients with multiple osteomas should be screened for Gardner’s syndrome, a genetic disorder associated with multiple osteomas and other abnormalities.

Histologically, osteomas consist of lamellar bone trabeculae surrounded by fibrofatty marrow. Their growth rate is highly variable; some remain stable for extended periods, while others grow up to 6 mm annually.

**Figure 8:**
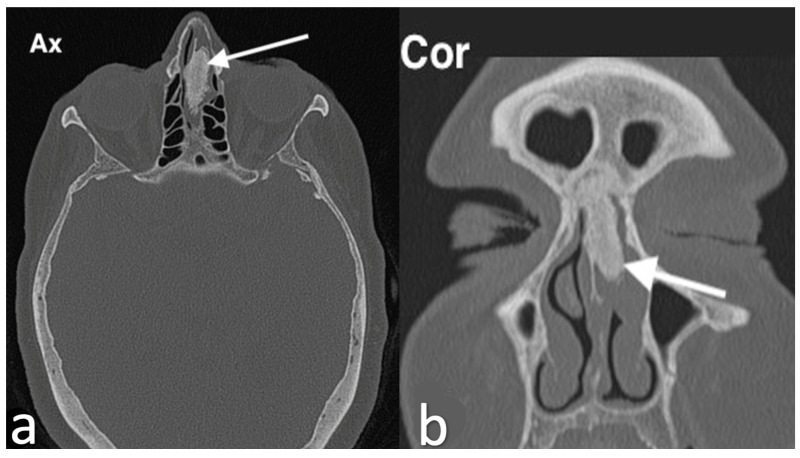
Osteoma as an incidental finding in a patient with rhinosinusitis. CT shows, in the upper anterior third of the nasal septum, an oval mass with smooth margin and bone density (arrow) visible on axial (**a**) and coronal (**b**) images. The mass extends into the left nasal cavity, partially obstructing the frontal recess.

Imaging studies show distinct features depending on the type of osteoma. On CT scans, osteomas typically present as areas with a ground-glass appearance surrounded by hyperdense regions, while MRI often shows enhancement (Figure 8). However, it can be challenging to distinguish osteomas from those with osteoblastic characteristics based solely on radiological findings, as these features may overlap.

Compact (ivory) osteomas typically present with a sessile base and are histologically characterized by dense bone with minimal marrow spaces and occasional Haversian canals. These lesions can range in size from a few millimeters to several centimeters.

In contrast, cancellous osteomas are often pedunculated and histologically resemble the bone from which they originate, featuring trabeculae of bone surrounded by fibrofatty marrow. Their surface may be either smooth or irregular, with cortical bone present along the periphery.

The primary treatment for osteomas is complete surgical excision, ensuring removal of the lesion at the interface with the cortical bone [30].

#### 3.2.6. Hemangiopericytoma

Sinonasal hemangiopericytoma (SNHPC) is an uncommon vascular tumor, mostly occurring in the low extremities, pelvic cavity and retro-peritoneum, with 15–17% found in the head and neck region. It predominantly affects middle-aged individuals and shows no strong gender predilection. Symptoms are generally nonspecific and may include nasal obstruction, epistaxis, breathing difficulties, sinusitis, headache, and nasal congestion. The nasal cavity is the most frequent site of occurrence, being twice as commonly affected as the paranasal sinuses, with the ethmoid and sphenoid sinuses being the most often involved [31].

These tumors are typically slow-growing over several years, often mimicking a benign process. However, they can invade adjacent structures such as the orbit and intracranial regions. Histological characteristics of HPC include uniform spindle-shaped cells with indistinct cytoplasm and prominent nuclei, arranged around vascular channels that commonly exhibit a branching, stag-horn pattern. On CT, SNHPC appears as a soft tissue mass that enhances following contrast administration and is frequently associated with bony destruction [32]. MRI typically shows these tumors as well-defined, solid masses with low to intermediate signal intensity on T1 images and heterogeneously high signal intensity on T2 images. A low-intensity rim, suggesting a pseudocapsule, may also be visible on T2 scans. Due to their high vascularity, the tumors often exhibit strong enhancement after contrast administration. Larger lesions may show hypointense flow voids, indicating blood vessels within the mass.

**Figure 9:**
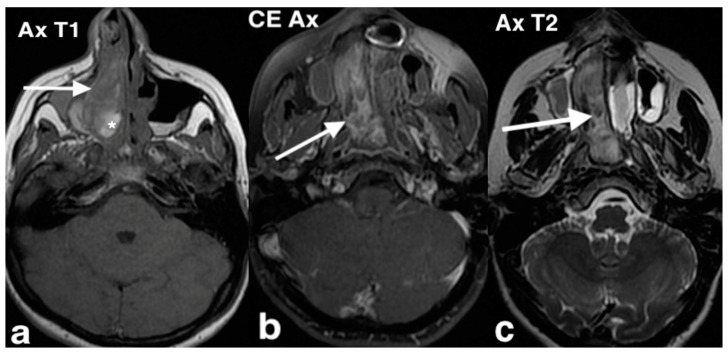
Hemangiopericytoma in a woman with epistaxis and bilateral nasal obstruction. On axial T1 MR image (**a**), the right nasal cavity is entirely occupied by an obstructing mass (arrow) with low–intermediate intensity and hyperintense hemorrhage areas (asterisk). Posteriorly, the mass, crossing the right choana, obstructs the nasopharynx lumen. On the axial fat saturation T1 after intravenous Gd image (**b**), the mass (arrow) is highly but inhomogeneously vascularized, with flow voids; on the axial T2 image (**c**), the mass (arrow) has medium–high intensity with an anterior low-intensity area, corresponding to hemorrhage; hypointense foci of flow void are also observed.

The differential diagnosis includes other highly vascularized lesions such as angiofibroma, glomus tumor, desmoid-type fibromatosis, and angioleiomyoma. Of particular interest in the described case were areas of high T1 signal and low T2 signal intensity, consistent with blood degradation products from previous hemorrhage. MRI is especially valuable for distinguishing tumor tissue from inflammatory fluid caused by sinus obstruction and for assessing possible extension to the skull base (Figure 9).

The primary treatment is wide local excision. For smaller tumors, endoscopic resection may be sufficient, provided the site of origin is clearly identifiable. SNHPC has a relatively low metastatic rate of 5–10%. However, given the potential for recurrence even years after initial treatment, lifelong follow-up is strongly recommended.

#### 3.2.7. Hemangioma

Hemangioma is a benign vascular tumor characterized by the abnormal proliferation of blood vessels. Within the nasal mucosa, hemangiomas are rare and typically manifest around the fourth decade of life, with no clear preference for either sex [33]. Histopathologically, they are classified into capillary, cavernous, hypertrophic, and mixed types. The capillary subtype is the most common and is often located on the anterior septal cartilage, especially during early childhood, where it may spontaneously regress. Conversely, cavernous hemangiomas are more prevalent in adulthood and are often linked to trauma [33].

Common symptoms of nasal hemangiomas include epistaxis, unilateral nasal obstruction, and rhinorrhea [33]. Histopathological analysis frequently reveals fibrocollagenous tissue with hemorrhagic areas, covered by a squamous epithelial lining. The tissue contains a network of thin-walled vascular channels, some of which are dilated and lined by flat epithelium [33]. These lesions are typically found in the bony septum or lateral nasal wall, although they are quite rare [34].

**Figure 10:**
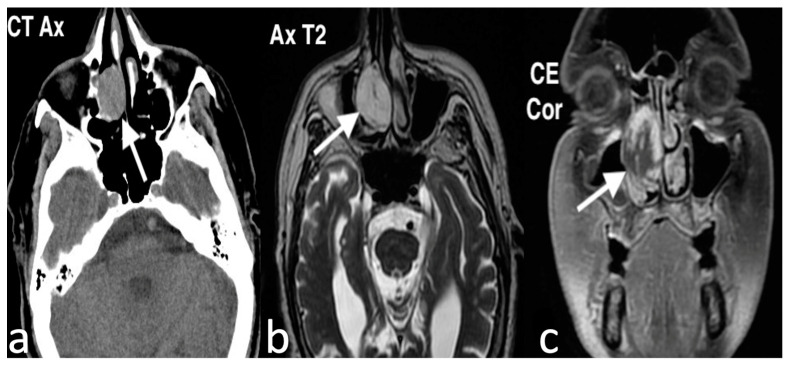
Hemangioma in a patient with right nasal obstruction and epistaxis. During rhinoscopy, a smooth dark-red mass was noted. The axial CT image (**a**) shows a well-defined, oval mass in the upper third of the right nasal cavity (arrow); on MR, the mass (arrow) occupies the middle–upper third of the right nasal cavity, showing high intensity on the axial T2 image (**b**); on coronal fat saturation T1 after intravenous Gd (**c**), the mass (arrow) shows intense heterogeneous enhancement.

Diagnostic imaging usually involves contrast-enhanced CT, which reveals a soft tissue enhancing mass, sometimes accompanied by bony destruction. CT is fundamental in assessing bone involvement and potential extension into nearby structures such as the paranasal sinuses or intracranial region [33]. On MRI, these lesions display intermediate signal intensity on T1 images and heterogeneous signal intensity with flow voids on T2 images. Areas of hemorrhage and pronounced enhancement following gadolinium contrast administration are often evident (Figure 10).

The differential diagnosis for nasal hemangiomas includes conditions such as venous hemangioma, angiofibroma, glomus tumor, lymphangioma, metastatic malignancies, hemangioendothelioma, Wegener’s granulomatosis, malignant nasal tumors, sinonasal papilloma and angiofibroma [34]. The primary treatment is surgical excision. Several techniques can be used, including traditional excision, laser ablation, cryotherapy, and electrocoagulation. Endoscopic excision is particularly effective, providing excellent results with no reported complications and superior cosmetic outcomes. For lesions with an arterial component or associated arteriovenous fistula, preoperative embolization using super-selective arteriography may be necessary.

#### 3.2.8. Antrochoanal Polyp

The antrochoanal polyp is the most common benign sinonasal tumor. It is a unilateral, solitary, inflammatory nasal polyp, more frequently observed in children than in adults. In pediatric patients, it accounts for up to 42% of all nasal polyps, while in adults, it represents 4–6%. Antrochoanal polyps are most frequently observed in individuals during their third to fifth decades of life, with a slight male predominance [35]. The exact cause of these polyps is unclear, although anatomical variations such as septal deviation, inferior turbinate hypertrophy, and concha bullosa are considered notable risk factors. In adults, chronic sinusitis is the most frequent underlying condition associated with their development [36].

Antrochoanal polyps typically originate in the maxillary sinus, extending through the antral or accessory ostium into the middle meatus, and may eventually reach the posterior choana and, in some cases, the nasopharynx. Common symptoms include nasal discharge, nasal blockage, snoring, epistaxis, and obstructive sleep apnea.

Histologically, these polyps are characterized by a lining of pseudostratified ciliated columnar epithelium, similar to the nasal mucosa. The underlying stroma consists of loose, edematous connective tissue. In children, these polyps often reflect an allergic process, as evidenced by eosinophilic infiltration, whereas in adults, they primarily result from inflammation, with minimal eosinophilic involvement [35].

Diagnosis typically begins with a CT scan, which reveals a hypodense mass originating from an enlarged and opacified maxillary sinus. Although antrochoanal polyps do not cause bony destruction, larger polyps may expand and widen an accessory ostium. Due to their soft consistency and location, they often exhibit a distinctive dumbbell shape.

**Figure 11:**
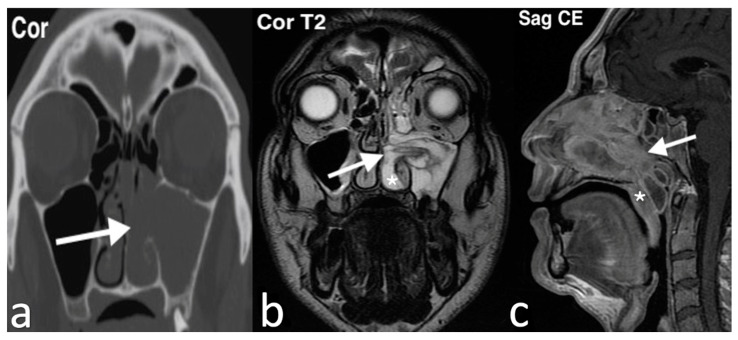
Antrochoanal polyp in a patient with left nasal obstruction and suspected rhinosinusitis. At rhinoscopy, a yellowish translucent lesion, arising from left ostiomeatal complex and extending up to the nasopharynx, is observed. The coronal CT image (**a**) shows a soft tissue mass filling the lumen of the left maxillary sinus and extending through an accessory ostium of the medial sinus wall in the adjacent nasal cavity (arrow); no evident bony erosion is visible. The MR coronal T2 image (**b**) shows a polypoid mass (arrow) with high intensity in the left maxillary sinus, extending through the middle meatus into the left nasal cavity between the inferior turbinate (asterisk), laterally displaced, and septum. Sagittal T1 after intravenous Gd image (**c**) shows peripheral enhancement of the mass (arrow), extending posteriorly toward the choana in the nasopharynx lumen, behind the soft palate (asterisk); note the mucosal thickening of sphenoid and frontal sinuses due to obstruction of ostiomeatal complex.

On MRI, antrochoanal polyps generally display signal characteristics similar to water, with low intensity on T1 images and high intensity on T2 images. However, polyps with proteinaceous content may show areas of increased T1 signal and intermediate-to-low T2 signal. After intravenous contrast administration, a thin rim of mucosal enhancement is typically visible, while the central portion of the polyp remains unenhanced (Figure 11) [36].

Surgery is the only definitive treatment for antrochoanal polyps. Complete removal of the mass, including its antral portion, is crucial to prevent recurrence, as simple polypectomy is associated with a high likelihood of regrowth. Historically, the Caldwell–Luc procedure was employed to excise the antral component; however, it has largely been abandoned due to risks such as facial paresthesia, infraorbital nerve injury, and interference with maxillary growth centers in children. Currently, the preferred approach is functional endoscopic sinus surgery (FESS), which is considered the gold standard.

#### 3.2.9. Lipoma

Lipoma is relatively common soft tissue tumor in adults, with the highest prevalence observed in individuals between the ages of 40 and 50. It is slightly more common in males. This benign and slow-growing tumor is histologically characterized by clusters of mature adipocytes, organized into lobules and separated by thin connective tissue septa. While 13% of lipomas are found in the head and neck region, the posterior neck, cheeks, tongue, floor of the mouth, and buccal sulcus are the most frequent locations [37]. Nasal cavity lipomas, however, are exceedingly rare and are predominantly reported in children as solitary masses. Cases involving the nasopharynx, nasal vestibule, nasal dorsum, and inferior turbinate have been documented but are uncommon [37].

The exact cause of lipomas remains unclear, though certain subtypes—such as conventional lipomas, spindle cell lipomas, and pleomorphic lipomas—appear to have a genetic basis and may be hereditary. Solitary lipomas are more frequently observed in females, while multiple lipomas (lipomatosis) are more common in males. Hereditary multiple lipomatosis, an autosomal dominant condition, is also predominantly seen in men.

**Figure 12:**
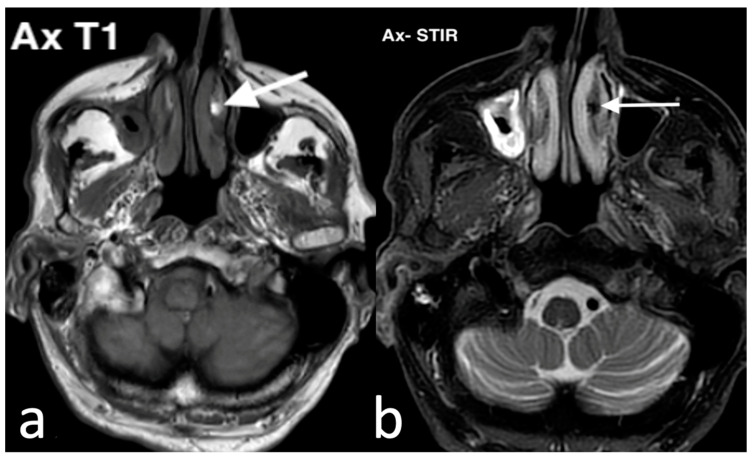
Lipoma in the inferior left nasal turbinate as an incidental finding on MRI. On axial T1 image (**a**), a millimetric oval spot in the inferior left nasal turbinate with high intensity (arrow) is observed; on axial STIR image (**b**), lipoma typically shows signal drop (arrow) due to fat signal suppression.

Patients with nasal cavity lipomas may remain asymptomatic or present with symptoms resembling those caused by other masses in the nasal cavity and sinuses. These may include nasal obstruction, facial swelling, tenderness, rupture, or bleeding. On plain CT imaging, lipomas typically appear as homogeneous, low-density, and often non-encapsulated masses. MRI provides additional detail and is valuable for assessing potential intracranial involvement. Lipomas exhibit high signal intensity on both T1 and T2 MRI images, show signal loss on fat-suppressed sequences, and do not enhance following intravenous gadolinium administration, unlike the surrounding nasal mucosa (Figure 12).

Treatment for nasal cavity lipomas involves simple surgical excision, which is curative and typically sufficient for resolving symptoms.

#### 3.2.10. Sinonasal Tract Angiofibroma

Sinonasal tract angiofibroma (STA) is a benign but highly vascular and locally destructive mesenchymal tumor. While commonly known as juvenile nasopharyngeal angiofibroma (JNA), this tumor is now officially termed sinonasal tract angiofibroma in the fifth edition of the World Health Organization Classification of Head and Neck Tumors. STA accounts for up to 0.5% of all head and neck tumors and predominantly affects young men, with a typical onset around 15 years of age (ranging from 10 to 18 years) [38]. It comprises less than 0.05% of all head and neck tumors. Although STA primarily affects males, rare cases have been documented in females and older males [39].

Globally, only six cases of histologically confirmed nasopharyngeal angiofibromas in adult males have been reported, often with a noticeable two-peak age distribution. In older patients, the disease is often more aggressive, exhibiting widespread local invasion, whereas younger individuals typically present with tumors that are more localized and less invasive [40]. STA has the potential to erode nearby anatomical structures, including bones, and may extend intracranially. Malignant transformation is extremely rare. The tumor typically originates from the sphenopalatine foramen, extending into the nasal cavity, nasopharynx, and PPF, often involving the pterygoid base [39].

Common symptoms include nasal obstruction and epistaxis, with additional signs like purulent nasal discharge, facial pain due to sinus drainage blockage, and conductive hearing loss from Eustachian tube obstruction [39,41]. Nasal endoscopy often reveals a hypervascular, lobulated mass with a smooth surface, typically protruding behind the middle turbinate, obstructing the choana, or even filling the entire nasal cavity. In some cases, proptosis and facial swelling may be observed due to invasion into the orbital and infratemporal fossae [41]. The histological characteristics of STA consist of two primary components: vascular spaces and a fibrous or collagenous stroma. The vascular spaces vary in size and shape, ranging from dilated branching vessels of varying thickness to narrow, slit-like capillaries. The fibrous or collagenous stroma contains fibroblasts, which may appear in spindle, round, or stellate forms [39]. The central area of the tumor is typically more cellular, composed mainly of fibroblasts or myofibroblasts. The stroma itself can be fibrous, edematous, or collagenized, and fibrinous thrombi may occasionally be seen within the dilated vessels [41]. Additionally, the stroma often contains an abundant number of mast cells. Despite the cellularity of the tumor, mitotic figures are generally absent, highlighting its benign nature [39,40,41].

Diagnosis is based on characteristic clinical and imaging features, with tissue biopsy generally avoided due to the risk of significant bleeding. On CT, STA appears as a highly vascularized lesion centered on the pterygopalatine fossa (PPF), with bony involvement that can be observed in three patterns: (1) expansion, thinning, and displacement of bone (e.g., anterior displacement of the posterior maxillary boundary, known as the Holman–Miller sign), (2) invasion into cancellous bone (e.g., pterygoid base), and (3) bone resorption and destruction. MRI is more effective than CT in evaluating intracranial, orbital, and cavernous sinus involvement [41]. The tumor typically appears isointense to muscle on T1 and hyperintense on T2 images, with flow-void signals due to enlarged blood vessels and intense enhancement after intravenous contrast administration [39].

**Figure 13:**
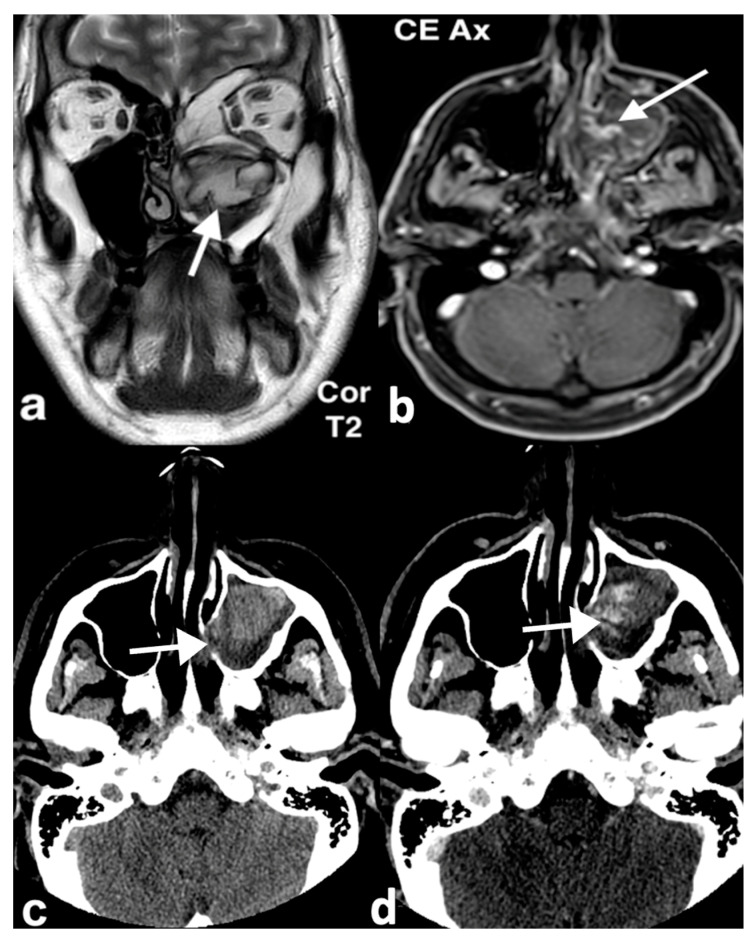
Sinonasal tract angiofibroma in a 43-year-old patient with recurrent epistaxis and left nasal obstruction, anosmia, left cheek pain, and headache for approximately one year. MRI: coronal T2 image (**a**) shows a lobulated mass (arrow) with peripheral low-intensity rim, centered at the left maxillary sinus and extending through an accessory ostium into the left nasal cavity, totally obstructed; on the axial fat saturation T1 after intravenous Gd image (**b**), the mass (arrow) shows heterogeneous enhancement with contrast media blush in the left nasal fossa, suggesting active bleeding. On axial CT (**c**), the mass (arrow) has a soft tissue density, showing (**d**) intense but heterogeneous enhancement (arrow) after intravenous iodinated contrast medium administration.

MRI plays a crucial role in detecting tumor extension into the intracranial, dural, and intracavernous regions. Dural contrast enhancement on post-contrast T1 images is a key indicator of dural invasion, while contrast-enhanced Fluid Attenuated Inversion Recovery (FLAIR) sequences may be more sensitive in detecting leptomeningeal spread (Figure 13) [39]. The distinguishing pattern of spread is vital for differentiating STA from other vascular and invasive lesions in the pediatric skull base, such as schwannomas, meningiomas, hemangiomas, hemangiopericytomas, and sarcomas [41].

The primary goal of surgical treatment is complete tumor removal with minimal morbidity. In some cases, preoperative selective embolization is used to reduce intraoperative blood loss and minimize associated risks. Surgical approach selection depends on the tumor’s extent. For years, external approaches, such as transpalatal techniques, Le Fort I osteotomies, lateral rhinotomy, midfacial degloving, facial translocation, and anterior craniofacial and lateral infratemporal/subtemporal approaches, were commonly employed. However, these external methods are associated with higher morbidity due to the extensive osteotomies required, which can lead to increased blood loss, prolonged operative time, and potential interference with normal facial growth in adolescent patients [41]. Alternatively, an endoscopic approach, typically reserved for early stage STA confined to the nasal cavity, nasopharynx, ethmoid, and sphenoid sinuses with minimal extension into the PPF, offers advantages. It avoids facial incisions, osteotomies, and bone plating, reducing the risk of craniofacial alterations in young patients [41].

### 3.3. Malignant Neoplasms

#### 3.3.1. Non-Hodgkin Lymphoma

Sinonasal malignancies are generally categorized into epithelial and non-epithelial types. Among these, Non-Hodgkin lymphoma (NHL) is the second most common malignancy in this area, following squamous cell carcinoma (SCC) [42], with recent imaging studies showing regional lymphadenopathy in 7–19% of cases [43]. Diffuse large B-cell lymphoma (DLBCL) and extranodal natural killer/T-cell lymphoma (ENKTL) are the most common subtypes of NHL found in the sinonasal region. Due to the limited anatomical space within the nasal cavity and paranasal sinuses, these tumors often develop insidiously, with their symptoms—such as nasal congestion, localized pain, and nosebleeds—being nonspecific and easily mistaken for other, more common conditions [42]. CT is the best technique to evaluate bone; bone destruction and invasion of adjacent structures may be observed with lytic or permeative bone destruction and bone remodeling on imaging. ENKTLs typically arise in the nasal cavity, presenting with poorly defined margins, internal necrosis, heterogeneous signal intensity, and significant enhancement of the solid portion on MRI. In contrast, DLBCLs are more frequently found in the paranasal sinuses, exhibiting homogeneous intensity on MRI, mild enhancement, a pattern of septal enhancement, and potential involvement of the intracranial or orbital areas [44].

**Figure 14:**
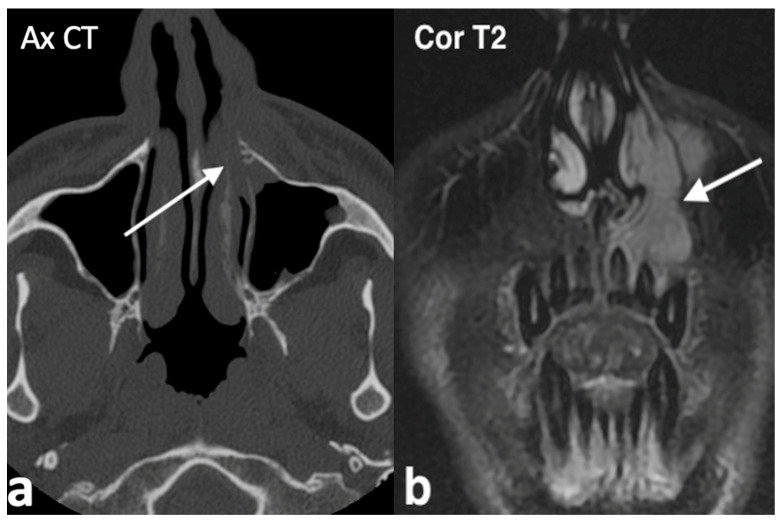
Non-Hodgkin lymphoma in a patient with monolateral left nasal obstruction. Rhinoscopy highlights a mass with a regular smooth surface responsible for raising mucosal lining of nasal floor. The axial CT image (**a**) shows a lobulated thickening of mucosal lining of the left lateral nasal wall (arrow), also extending to the maxillary sinus; note that lymphoid tissue permeates the adjacent cortical bone of the medial wall of the maxillary sinus. On the MRI coronal T2 fat saturation image (**b**), the lesion (arrow) shows high intensity, and extends into the lateral aspect of the left inferior turbinate, invading the left half of maxillary bone.

MRI has proven to be particularly effective in diagnosing sinonasal lymphomas, which typically present as masses with soft tissue attenuation. Specifically, NHLs generally appear isointense on T1 images and hyperintense on T2 images (Figure 14). In cases involving the maxillary sinus, NHLs often show a lower ADC, a feature that can help distinguish them from SCC. In a recent study, dynamic contrast-enhanced MRI (DCE-MRI) and DWI were able to detect differences between SL and SCC. The peak enhancement (EP), time to peak (TTP), maximum contrast enhancement ratio (MCER), and ADC values showed significant differences when compared. In contrast to the SCC group, the SL group exhibited a longer TTP along with lower values for EP, MCER, ADC, and relative ADC (rADC). This suggests that SCC demonstrates a rapid uptake and clearance of the contrast medium, leading to a higher EP and a shorter duration of enhancement. Treatment for sinonasal non-Hodgkin lymphoma (SN-NHL) varies depending on the subtype and stage of the disease, often requiring a combination of therapies, including chemotherapy and/or radiation therapy. For sinonasal diffuse large B-cell lymphoma (SN-DLBCL), there is no established consensus on an optimal treatment strategy. However, patients treated with chemoradiotherapy tend to achieve better overall survival compared to those receiving either multi-agent chemotherapy or radiation therapy alone.

The addition of immunotherapy may enhance treatment regimens, improving progression-free and overall survival when combined with multi-agent chemoradiotherapy. The current standard treatment for SN-DLBCL commonly involves R-CHOP (rituximab, cyclophosphamide, doxorubicin, vincristine, and prednisone) combined with radiation therapy. Radiation may also be an effective option for localized disease [45].

#### 3.3.2. Esthesioneuroblastoma

Esthesioneuroblastoma (ENB), also known as olfactory neuroblastoma, is a rare and aggressive malignant tumor of the sinonasal tract that arises from the olfactory neuroepithelium. This neoplasm is known for its local invasiveness and potential to metastasize through both hematogenous and lymphatic pathways. ENB represents approximately 2% to 6% of malignancies in the nasal cavity and paranasal sinuses and accounts for about 0.3% of all cancers of the upper aerodigestive tract [46].

The disease exhibits a bimodal age distribution, with peaks in the second and sixth decades of life, and it affects males and females equally. However, rare cases have been documented in children under 10 years old [47]. ENB is believed to originate from specialized olfactory neuroepithelial cells located in the superior regions of the nasal cavity, including the upper nasal septum, the superior nasal concha, the roof of the nasal cavity, and the cribriform plate of the ethmoid sinus.

Clinically, ENB often presents with nonspecific symptoms resembling benign inflammatory or infectious conditions, leading to diagnostic delays. Early signs such as nasal obstruction and epistaxis are common, though symptoms may vary depending on the tumor’s size and location. For example, anosmia, as observed in some patients, can precede the diagnosis by years [46,47].

**Figure 15:**
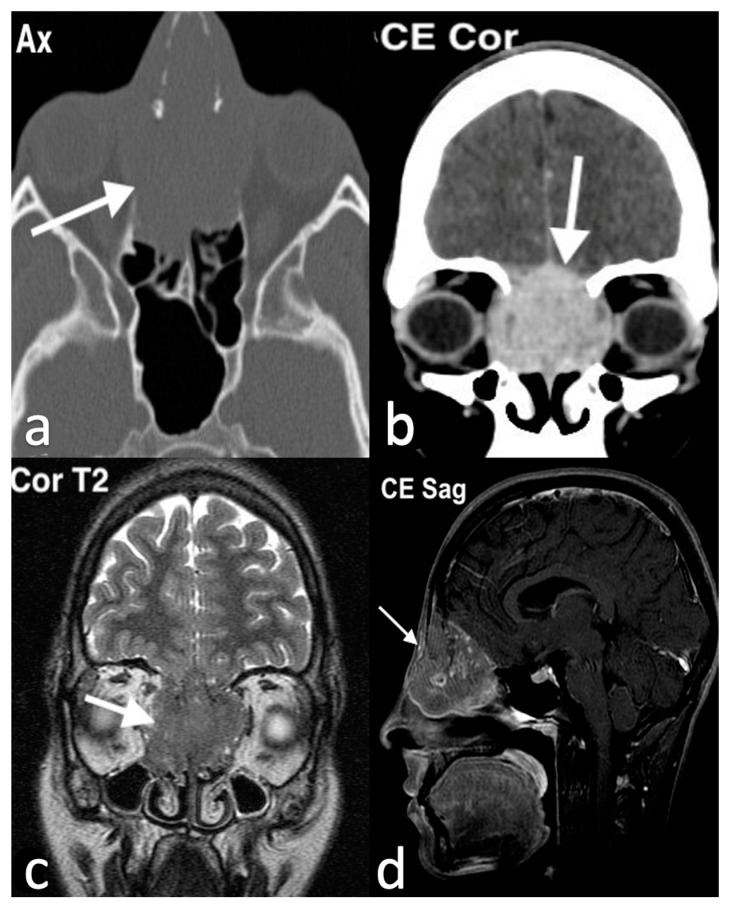
Esthesioneuroblastoma in a 22-year-old girl with headache worsening in the supine position, associated with serous rhinorrhea and anosmia. She also had pain at the root of the nasal pyramid (widened and deformed) and eyelid edema. Endoscopically, at the level of the olfactory slit, an oval mass with a smooth surface and yellowish color, covered by a dense vascular reticule, was bilaterally evident. On the axial CT image (**a**), a mass (arrow) in both nasal cavities, with marked erosion of ethmoid and nasal bones, is visible; on the contrast-enhanced coronal CT image (**b**), the mass (arrow) shows intense and inhomogeneous enhancement, with massive erosion of ethmoid, involving the medial wall of the orbits and cribriform plate. MRI: on the coronal T2 image (**c**), the mass (arrow) shows low–intermediate intensity, with invasion of extraconic orbital fat; transcompartmental extension is also visible with intracranial extension in the olfactory groove; on the sagittal fat saturation T1 after intravenous Gd image (**d**), the tumor (arrow) shows heterogenous enhancement.

Imaging plays a key role in the diagnosis of ENB. On CT scans, ENB often appears as a homogenous mass with necrotic non-enhancing regions and may demonstrate bony erosion. MRI, on the other hand, is more effective in assessing the extent of soft tissue involvement. A characteristic finding is a dumbbell-shaped mass spanning the cribriform plate, with its waist located at the cribriform plate, the superior portion extending into the anterior cranial fossa, and the inferior portion into the nasal cavity. Peritumoral cysts at the tumor–brain interface are another imaging hallmark. Contrast-enhanced MRI is particularly useful in differentiating the tumor from obstructed secretions in the paranasal sinuses and in detecting meningeal invasion, extradural involvement, and perineural spread (Figure 15) [47].

There is no universally accepted staging system for ENB due to its rarity and the diversity of histologic subtypes among sinonasal malignancies. The first classification system, introduced by Kadish in 1976, categorized tumors into three groups based on their anatomical extent: **Group A**, confined to the nasal cavity; **Group B**, extending into the paranasal sinuses; and **Group C**, spreading beyond the paranasal sinuses. In 1992, Dulgerov and Calcaterra proposed a more detailed staging system based on the TNM classification, incorporating factors such as cervical lymph node involvement and distant metastases, which can be assessed using imaging studies [48].

Treatment strategies for ENB typically involve a multimodal approach, combining surgery, radiation therapy, and chemotherapy. For small, localized tumors without evidence of regional or distant metastasis, surgical excision is the primary treatment. In cases of more advanced disease, extensive surgical resection—often a craniofacial procedure performed via open or endoscopic-assisted techniques—is combined with postoperative radiotherapy to improve local control. Preoperative radiotherapy is an alternative approach with comparable outcomes. Additionally, induction chemotherapy or concurrent postoperative chemoradiation may enhance local, regional, and distant disease control [49].

#### 3.3.3. Intestinal-Type Adenocarcinomas

Sinonasal intestinal-type adenocarcinoma (ITAC) represents 8–25% of all the sinonasal malignancies, predominantly affecting men between the ages of 55 and 60 years. This type of cancer is particularly common among workers in the hardwood industry. The typical latency period between initial exposure and the development of adenocarcinoma in the sinonasal tract is around 40 years [50,51].

Sinonasal adenocarcinomas generally originate from the following sites: ethmoid sinuses (40%), nasal cavity (28%), maxillary antrum (23%), and other indeterminate locations (9%) [52]. Tumors related to occupational exposure primarily affect men and tend to originate in the ethmoid sinuses. In contrast, sporadic tumors, which are more common in women, often arise in the maxillary sinuses. Patients with sporadic tumors typically experience shorter survival compared to those with occupational-exposure-related tumors. Maxillary sinus tumors often remain asymptomatic until they reach advanced stages, while tumors in the nasal cavity and ethmoid sinuses usually become symptomatic earlier, before invading local structures.

Common symptoms at presentation include nasal obstruction, epistaxis, hyposmia, cheek masses, exophthalmos, rhinorrhea, and facial nerve involvement. Upon examination with fiberoptic endoscopy, ITAC typically appears as a soft tissue mass, often accompanied by bleeding [53].

In terms of morphology, ITAC resembles adenocarcinoma or adenoma of the small intestine, or even normal intestinal mucosa. Histologically, ITACs can present in five distinct morphologic patterns: papillary, colonic, solid, mucinous, and mixed.

**Figure 16:**
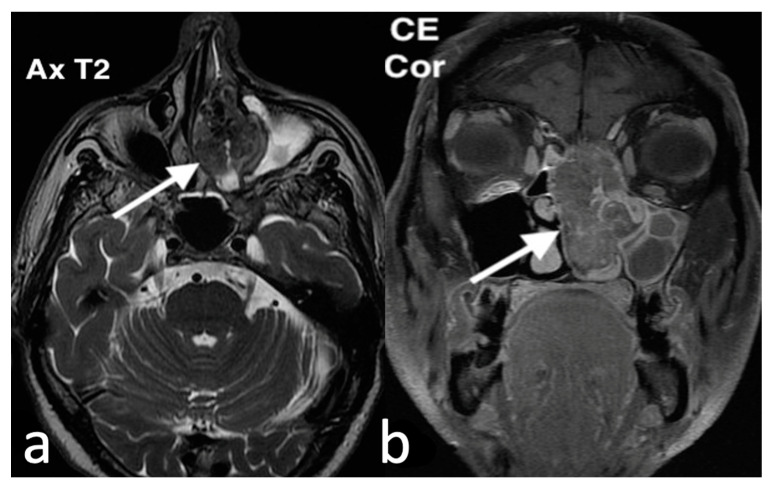
Sinonasal intestinal-type adenocarcinoma in a patient with left nasal obstruction, epistaxis and headache. During rhinoscopy, an irregular-surfaced mass with high tendency to bleed was observed. MRI: the axial T2 image (**a**) shows a lobulated mass (arrow), in the left nasal cavity, obstructed, which expands the contiguous bony structure and medially erodes the nasal septum, invading the contralateral right nasal cavity; the mass causes obstruction of the ostiomeatal complex; mucocele of the left maxillary antrum is also visible; the coronal T1 after intravenous Gd image (**b**) shows heterogenous enhancement of the mass (arrow).

CT imaging is useful in detecting bone involvement and erosion, offering insights into the tumor’s aggressiveness and extent of invasion into adjacent structures. MRI is more effective in evaluating the overall size of the tumor and its interaction with neighboring structures, including any perineural spread [52]. The MRI appearance of adenocarcinomas varies depending on their mucin content, cellularity, and the presence of hemorrhage or necrosis. Mucin-producing adenocarcinomas typically show spontaneous hyperintensity on T2 images, with possible high signal intensity on T1 images in areas of hemorrhage, along with slight enhancement (Figure 16) [53].

When diagnosing masses at the anterior skull base with intracranial extension, it is important to consider differential diagnoses such as squamous cell carcinoma, lymphoma, esthesioneuroblastoma, sinonasal melanoma, metastasis, and aggressive infections, particularly fungal infections.

The primary treatment for ITAC, whenever feasible, is radical surgical resection. Transnasal endoscopic surgery (TES) has become the preferred approach for many sinonasal cancers, particularly those located in the nasoethmoidal complex. For tumors that involve or extend into the anterior skull base, combined surgical approaches, such as craniofacial or cranioendoscopic resection, or endoscopic resection with transnasal craniectomy, are required to achieve clear margins.

Radiotherapy is generally used as an adjuvant treatment for high-grade or advanced-stage tumors [53]. ITAC is often diagnosed at an advanced stage, and the most common sites of metastasis include the lungs, liver, and bones. As a result, the prognosis is generally poor, with high relapse rates even after radical surgery and radiotherapy. The five-year survival rate ranges from 25% to 45%.

#### 3.3.4. Melanoma

Primary sinonasal mucosal melanoma (SNM) is a rare and highly aggressive malignancy, accounting for less than 4% of all head and neck cancers and 55% of all head and neck melanomas. It is strongly associated with smoking. SNM typically originates from melanocytes in the mucosa of the nasal cavity (about 50% of cases) and paranasal sinuses (20%). The most common sites of involvement are the nasal septum, the lateral wall of the nasal cavity, and the inferior nasal concha [54]. It is important to differentiate primary SNM from secondary tumors originating from cutaneous or uveal melanomas.

The clinical presentation of SNM is nonspecific, with common symptoms including epistaxis, nasal obstruction, and headache [54]. Histologically, SNM typically shows a proliferation of melanocytes, both as individual cells and nests, starting within the basal layer. Ulceration is a common feature, and there is often pagetoid involvement of the epidermis. These lesions tend to be asymmetric, exhibiting a lack of maturation and cytological atypia. The melanoma cells are frequently large and epithelioid, with abundant eosinophilic cytoplasm. Around 50% of mucosal melanomas are amelanotic or exhibit minimal pigmentation, which can create overlapping features and pose diagnostic challenges when distinguishing mucosal melanomas from other small cell or undifferentiated sinonasal tumors [54].

On CT, SNM usually appears as a nonspecific soft tissue mass. However, CT is crucial in treatment planning, as it helps assess the integrity of regional bony structures, such as the sinonasal and orbital walls, as well as the carotid and optic canals, and skull base [55]. MRI findings for SNM can vary depending on the amount of melanin present, with some cases being amelanotic. Melanin-rich melanomas show a hyperintense signal on unenhanced T1 images compared to muscle, and a hypointense signal on T2 sequences. On the other hand, amelanotic melanomas appear isointense to muscle on T1 images.

After intravenous gadolinium administration, SNM typically exhibits rapid enhancement with minimal washout. Diffusion-weighted imaging (DWI) usually shows restricted diffusion, with low values on the ADC map. For evaluating residual or recurrent SNM and assessing distant metastases, 18F-FDG PET-CT is valuable [54].

**Figure 17:**
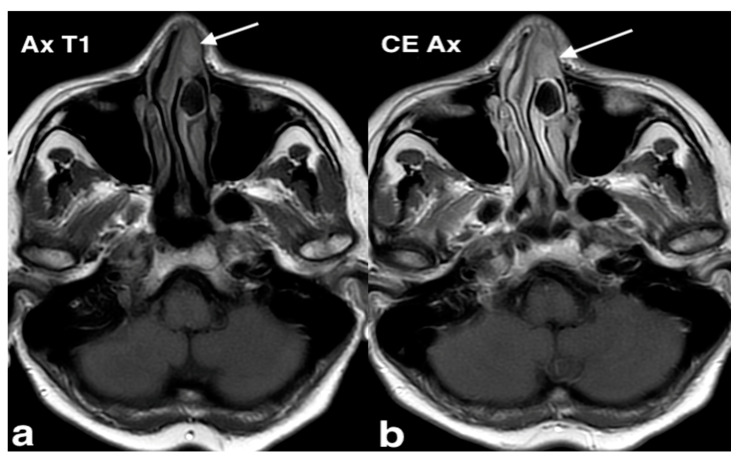
Melanoma in a 67-year-old woman with left nasal obstruction. On axial T1 image (**a**), a medium–high-intensity oval formation (arrow) in the left antero-superior nasal fossa is appreciable; on the axial T1 after intravenous Gd image (**b**), the mass (arrow) shows inhomogeneous enhancement.

Differential diagnoses for T1-hyperintense sinonasal lesions include hemorrhagic metastases and primary lesions such as hemangioma, lymphangioma, sinonasal tract angiofibroma, and fungal infections. For amelanotic melanoma, MRI characteristics are nonspecific and can resemble other malignancies like squamous cell carcinoma, adenocarcinoma, minor salivary gland tumors, inverted papilloma, and neuroblastoma. Plasmacytoma and fibro-osseous lesions may also present similarly on MRI, as can chondrosarcoma, although CT can help identify calcified matrix (Figure 17) [55].

In metastatic cases, melanoma may lose its melanocyte-specific markers, resulting in unusual morphology and immunohistochemical features resembling solitary fibrosus tumors (SFTs) [56].

The prognosis for sinonasal melanoma is generally worse than that for nasal melanoma, likely due to delayed diagnosis. SNM is extremely aggressive, with a recurrence rate of up to 64% within the first year after surgery [54,55].

#### 3.3.5. Rhabdomyosarcomas

Rhabdomyosarcoma (RMS) is a rare soft tissue cancer that arises from myogenic cells, primarily affecting the pediatric population, especially those under 20 years of age [57,58]. When it occurs in the paranasal sinuses, RMS accounts for 10% to 15% of head and neck RMS cases in adults [57]. Based on the tumor’s location, RMS in the head and neck region is categorized into three main subtypes: orbital, non-orbital parameningeal (including the nasopharynx/nasal cavity, middle ear, paranasal sinuses, and the infratemporal fossa/pterygopalatine space), and non-orbital non-parameningeal. The most common histological subtype is embryonal, followed by alveolar (which is most commonly found in paranasal locations), botryoid, pleomorphic (more frequent in adults), and unclassified. Among these, alveolar RMS is the most aggressive subtype, with a higher recurrence rate and lower overall survival [58].

In the head and neck region, RMS typically presents with nonspecific symptoms resulting from the local mass effect. These can include headache, nasal congestion, unilateral epistaxis, and otorrhea, which may mimic benign conditions [57,58,59].

**Figure 18:**
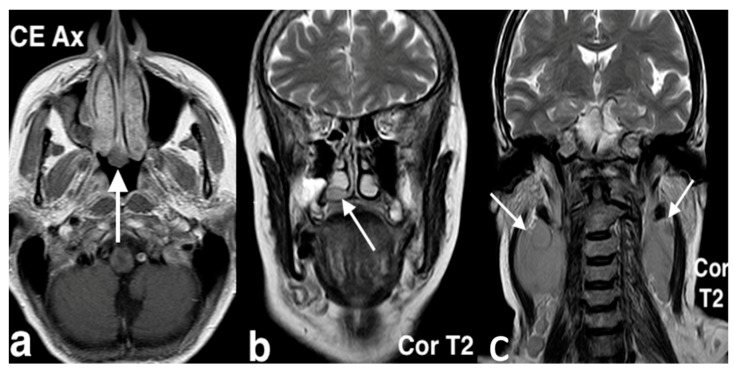
Rhabdomyosarcoma in a 65-year-old woman with nasal rhinorrhea and epistaxis. MRI: the axial T1 after intravenous Gd image (**a**) shows a nodular tumor (arrow) with low vascularity, attached and eroding the third posterior part of the nasal septum; on the coronal T2 image (**b**), the mass (arrow) with intermediate intensity is located in the right nasal fossa floor, between the inferior turbinate and nasal floor; the coronal T2 image (**c**) shows bilaterally metastatic lymph nodes in II-III levels (arrows).

Histologically, RMS shows cellular round cells characterized by large clusters, nests, cords, and trabeculae of primitive round cells, which are separated by fibrovascular septa of varying thickness [58]. These cells display hyperchromatic nuclei, often with small, conspicuous nucleoli. Multinucleated tumor giant cells, commonly arranged in a wreath-like fashion, are frequently observed. Additionally, round-to-oval rhabdomyoblasts with abundant eosinophilic cytoplasm may be present. Tumors typically exhibit brisk mitotic activity and may show variable necrosis [58]. On CT scans, RMS usually appears as a soft tissue mass in the nasal cavity, with a relatively homogeneous texture and solid enhancement following intravenous contrast administration. MRI shows a heterogeneous signal that is either equal to or more intense than muscle, making it especially useful for assessing tumor extension in paranasal locations, as well as the pterygopalatine and infratemporal fossae (Figure 18) [57,59].

In adults, RMS tends to have a significantly higher rate of distant metastasis compared to pediatric cases; so, PET scanning is strongly recommended. Endoscopy and biopsy are crucial for confirming the diagnosis. However, there are no specific endoscopic features for RMS, apart from the embryonal subtype, which may resemble a gelatinous nasal polyp [58,59].

The preferred treatment for children typically includes surgical excision, followed by radiotherapy and chemotherapy. However, complete radical excision is not always possible due to the tumor’s location near vital structures. In adults, the aggressive and invasive nature of RMS often requires alternative management approaches [59]. Radiotherapy may be considered as a therapeutic option due to the radiosensitivity of adult RMS.

Prognostic factors that indicate a better outcome include being under 20 years of age, having a tumor smaller than 5 cm in its largest dimension, the absence of distant metastases, and achieving negative surgical margins [59].

## 4. Conclusions

Lesions of the nose are among the most challenging conditions for otolaryngologists to diagnose and treat. Radiological imaging plays a critical role in both the diagnostic and therapeutic pathways of sinonasal tumors. It not only facilitates the initial diagnosis but also helps define the extent of disease, guides surgical planning, and monitors response to therapy. Computed tomography (CT) excels in assessing local bone invasion, identifying intralesional calcifications, and visualizing feeding arteries. Characteristic bone invasion patterns include extensive destruction associated with high-grade malignancies and slow, expansile growth typical of benign lesions or low-grade malignancies. MRI, on the other hand, provides superior contrast resolution, allowing for differentiation of soft tissue components of tumors, detection of perineural spread, and assessment of dural invasion. These imaging features guide the determination of pre-treatment prognosis, the selection of therapeutic modalities, and tumor staging. Nasal endoscopy with biopsy remains the gold standard for diagnosis. Our review offers radiologists, trainees, and clinicians a visual learning resource, often emphasizing the characteristic imaging findings of diseases or conditions, as well as offering concise explanations that help in identifying diagnostic patterns. The utility of using various imaging modalities for a specific clinical scenario helps to compare their relative strengths and limitations in depicting pathological findings. In our review, we have also included a report on some rarer diseases, atypical presentations, and complex conditions, providing a valuable reference for conditions that may not be commonly encountered. Through systematic presentation of images, this review helps radiologists develop and refine their ability to recognize imaging patterns, differentiate between pathologies, and avoid common diagnostic pitfalls.

## Data Availability

No new data were created or analyzed in this study. Data sharing is not applicable to this article.
